# Limitations of standard memory assessments in breast cancer survivors

**DOI:** 10.1515/tnsci-2025-0401

**Published:** 2026-08-24

**Authors:** Meenakshie Bradley-Garcia, Annick F.N. Tanguay, Gabriela Haddad, Adelaide Jensen, Melanie J. Sekeres

**Affiliations:** School of Psychology, Faculty of Social Sciences, University of Ottawa, Ottawa, Canada

**Keywords:** breast cancer, cancer treatment, episodic memory, ecological validity, chemofog

## Abstract

**Objectives:**

Breast cancer is the most commonly diagnosed cancer in women worldwide, and treatments like chemotherapy are often linked to post-treatment cognitive impairments, including memory deficits. Standardized episodic memory tests may fail to capture the nuanced, real-world aspects of memory, potentially underestimating impairments in cancer survivors. This study seeks to investigate the impact of cancer treatment on episodic memory in breast cancer survivors (BCS) by comparing standard cognitive measures with ecologically valid measures of naturalistic memory, which may capture the subtle memory impairments.

**Methods:**

BCS at least 6 months post-treatment (n=45) and non-cancer control (NC; n=43), aged 30 to 65, completed two cognitive tasks to assess episodic memory (Taler Stories and Verbal Paired Associates, VPA) and two questionnaires to assess perceived cognitive workload (National Aeronautics and Space Administration Task Load Index) and perceived episodic and semantic memory abilities (Survey of Autobiographical Memory).

**Results:**

BCS recalled fewer verbatim and gist details for the Taler Stories than NC, with impairments in gist memory persisting during delayed recall. However, no memory deficits were evident on the VPA task in BCS compared to NC. BCS reported greater cognitive effort during the story recall tasks, but no group differences emerged for the VPA task. BCS also self-reported heightened subjective difficulties with episodic and semantic memory compared to NC, which was consistent with poorer performance on the Taler Stories.

**Conclusions:**

Impaired performance on complex naturalistic memory, despite similar VPA memory task performance, suggests that standard assessments may not fully capture everyday memory challenges faced by BCS.

## Introduction

Breast cancer is the most commonly diagnosed cancer among women worldwide, accounting for one in every four diagnoses in women [1]. Although cancer treatments have substantially improved survival rates [2], growing evidence highlights treatment-related cognitive side effects, particularly with chemotherapy [3]. Colloquially known as “chemobrain”, chemotherapy-related difficulties in memory, executive function, and processing speed [4] commonly emerge during or after treatment and may persist for years [5], 6]. These cognitive changes can hinder interpersonal, occupational, and everyday functioning, and ultimately reduce quality of life [7].

Memory impairment, including episodic memory, is frequently reported by breast cancer survivors (BCS) [8], 9]. Episodic memory involves the vivid recollection of personal events, including temporal, contextual, and perceptual details (e.g., recalling the last Olympic gold medal hockey game) [10]. While rich in detail, episodic memories can co-exist with gist-like versions that convey general event content but lack specific, vivid features [11], 12]. Both fine-grained episodic details and more coarsely-grained gist-like details depend on the hippocampus, engaging posterior and anterior regions respectively [13], [14], [15]. Over time, as a memory becomes less episodically-detailed and more schematized (generalized representation of a class of event; e.g., memory of a typical hockey game) [16], 17], its retrieval may become increasingly supported by the medial prefrontal cortex (mPFC) and less dependent upon detailed hippocampal representations [11], [18], [19], [20]. In contrast, semantic memory refers to general knowledge and facts (e.g., knowing each team has six players on the ice), and is not linked to a specific event [10]. Due to episodic memory’s reliance on the hippocampus for encoding and detailed retrieval, episodic memory is especially vulnerable to conditions that affect hippocampal integrity [18]. Consequently, individuals with medial temporal lobe damage often show impaired recollection of episodically-specific event details while relying on more generalized, mPFC-dependent schematic representations and semantic knowledge to support memory retrieval [12].

Numerous classes of chemotherapeutic drugs are neurotoxic to the hippocampus, causing structural (e.g., volume reduction, deformation, neurogenesis) [8], [21], [22], [23], [24] and functional (e.g., altered activity and connectivity) [23], 25], 26] hippocampal disruptions, offering potential mechanisms underlying chemotherapy-related episodic memory deficits [27]. Along the hippocampal long-axis, reduced posterior hippocampal volume predicted impoverished episodic autobiographical memory in breast cancer survivors [21], suggesting that episodically-detailed narrative memory is particularly vulnerable to impairment post-treatment, while more schematic representations may be less affected.

Conventional memory assessments used to assess episodic memory, including list learning and verbal paired associate tasks, though reliable, often lack ecological validity and may not reflect real-world cognitive functioning [28]. For instance, list-learning tasks assess immediate recall but lack everyday demands such as remembering conversations or personal events. BCS often show poorer performance on early list learning trials, suggesting initial encoding challenges that improve with repetition and familiarity [29], [30], [31]. However, some show delayed recall impairments despite intact immediate recall and recognition, suggesting deficits in retrieval rather than encoding deficits [6], 31]. Other studies report no differences between BCS and controls [32], 33], highlighting the complexity of cognitive impairments and the need for assessments that better characterize memory processing differences with a higher degree of sensitivity.

Unlike the arbitrary word pairs used in verbal paired associates tasks, narrative memory assessments engage more complex mnemonic processes including schematic and semantic representations, and require the construction and maintenance of complex temporal and contextual relational structures within an event [34]. Consequently, narrative memory tasks offer greater ecological validity and are more sensitive to detecting subtle memory disruptions across multiple mnemonic domains. Naturalistic memory tasks like narrative recall, better mimic real-life memory experiences and can evaluate precise verbatim recollection, gist-based recall, and memory distortions during recollection of complex events [35]. This is meaningful as verbatim memory typically declines with age, while gist memory remains more stable and less affected by hippocampal damage [35], 36]. Supporting this, a study using the Wechsler Memory Scale – Logical Memory (WMS – LM) story recall task found that BCS who were 5–15 years post-treatment initially recalled fewer narrative details than non-cancer controls (NC), although this difference resolved eight months following baseline [29]. By using unit-based scoring of narrative recollection, including verbatim, gist, and distortion sub-types, we can potentially detect more subtle memory impairments that may not be captured by dichotomous scoring (i.e., correct or incorrect) [37] used in standardized memory assessments like verbal paired associates and the original scoring for the WMS-LM, leading to an underestimate of cognitive impairment.

Compounding this challenge, BCS often experience subjective cognitive decline post-chemotherapy [9], even in cases which fail to show impairments using objective measures [38]. A potential variable contributing to this discrepancy is perceived cognitive workload, or the mental effort required to complete a task [39], which is influenced by cognitive reserve, task complexity, and neural efficiency [40]. In addition to the hippocampal neurotoxicity discussed above, chemotherapy treatment leads to broader structural and functional changes throughout the brain (e.g., prefrontal hyperactivation, decreased grey and white matter integrity, and alterations in the default mode network) [41], [42], [43], which may contribute to inefficient network processing during cognitive tasks. The mismatch between objective and subjective complaints by cancer survivors may reflect the need to engage energy-intensive neural compensatory mechanisms in order to effortfully perform at control levels [44], 45].

This study examined whether an ecologically valid measure of narrative event memory could identify subtle memory deficits that may not be captured by a standard episodic memory assessment. We hypothesized that BCS would recall fewer episodically rich (i.e., verbatim) details during recall of the Taler Stories relative to NC. Additionally, we expected BCS to exhibit more difficulty recalling word pairs during the first trial of the Verbal Paired Associates (VPA) task, with improvements across trials. Memory performance was predicted to be associated with higher cognitive effort reported by BCS.

## Materials and methods

### Participants

We recruited female BCS, aged 30 to 65, who were >6 months post-treatment, and age-matched NC. Recruitment was conducted through community outreach, social media advertisements, the University of Ottawa’s Integrated System of Participation in Research community pool, and the Cancer Foundation Maplesoft-Jones Centre at the Ottawa Regional Cancer Foundation. Exclusion criteria included: a mental health diagnosis and/or initiating mood-altering medications within <12 months, a lifetime diagnosis of a neurodevelopmental or neurological disorder, current substance dependence or cannabis use >7 times/month, non-fluency in English, residence outside of Canada, and a Montreal Cognitive Assessment (MoCA) score ≤21 [46]. Of the ninety-three eligible participants, two withdrew (BCS, n=2) and three were excluded due to audio recording failures (BCS, n=2; NC, n=1), resulting in a sample of 88 participants (BCS, n=45; NC, n=43; Tables 1 and 2). The study was approved by the University of Ottawa’s Research Ethics Board (H-04-21-6706) and adhered to the Tri-Council Policy Statement: Ethical Conduct for Research Involving Humans.

**Table 1:** Participant demographics.

	BCS (n=45)	NC (n=43)
	Mean (SD) or % (n)	Mean (SD) or % (n)
**Age**	51.22 (8.73)	49.88 (10.67)
**Race**		

White	95.6 % (43)	81.4 % (35)
Black	0.0 % (0)	2.3 % (1)
East Asian	0.0 % (0)	2.3 % (1)
South Asian/East Indian	2.2 % (1)	2.3 % (1)
Indigenous	0.0 % (0)	2.3 % (1)
Latin American	0.0 % (0)	2.3 % (1)
Person of mixed origin	0.0 % (0)	2.3 % (1)
Turkish	2.2 % (1)	0.0 % (0)
West Asian, North African, Arab	0.0 % (0)	2.3 % (1)
Prefer not to answer	0.0 % (0)	2.3 % (1)

**Province of residence**		

Alberta	0.0 % (0)	4.7 % (2)
British Columbia	4.4 % (2)	9.3 % (4)
Manitoba	8.9 % (4)	0.0 % (0)
New Brunswick	2.2 % (1)	0.0 % (0)
Nova Scotia	4.4 % (2)	0.0 % (0)
Northwest Territories	4.4 % (2)	0.0 % (0)
Ontario	55.6 % (25)	69.8 % (30)
Prince Edward Island	2.2 % (1)	0.0 % (0)
Quebec	13.3 % (6)	11.6 % (5)
Saskatchewan	4.4 % (2)	2.3 % (1)
Yukon	0.0 % (0)	2.3 % (1)

**Completed education level**		

High school diploma	6.7 % (3)	2.3 % (1)
College degree	24.4 % (11)	18.6 % (8)
Bachelor’s degree	44.4 % (20)	39.5 % (17)
Master’s degree	17.8 % (8)	27.9 % (12)
Doctorate degree	2.2 % (1)	7.0 % (3)
Other	4.4 % (2)	4.6 % (2)

**Income**		

<$30,000	0.0 % (0)	7.0 % (3)
$30,000–$50 000	2.2 % (1)	9.3 % (4)
$50,000–$100,000	24.4 % (11)	20.9 % (9)
$100,000–$200,000	46.7 % (21)	39.5 % (17)
>$200,000	20.0 % (9)	11.6 % (5)
I prefer not to answer	6.7 % (3)	11.6 % (5)
**Current substance use**	46.7 % (21)	60.5 % (26)

**Drug-frequency**		

Daily	20.0 % (9)	27.9 % (12)
2–3 times per week	8.9 % (4)	11.6 % (5)
Once per week	2.2 % (1)	9.3 % (4)
Once per month	22.2 % (10)	23.3 % (10)
Not applicable	46.7 % (21)	27.9 % (12)
**Mental health diagnosis (lifetime)**	13.3 % (6)	16.3 % (7)
**Mental health symptoms (past 12 months)**	0.0 % (0)	4.7 % (2)
**Mental health symptoms (past 6 months)**	0.0 % (0)	0.0 % (0)
**Medications**	82.2 % (37)	51.2 % (22)
**Mood altering medications**	13.3 % (6)	9.3 % (4)
**Menopausal status**	82.2 % (37)	53.5 % (23)

BCS, breast cancer survivors; NC, non-cancer controls; n, sample size; SD, standard deviation; %, percentage.

**Table 2:** Breast cancer diagnosis and treatment details.

	%, (n)
**Time since diagnosis**	

1–2 years ago	17.8 % (8)
2–5 years ago	48.9 % (22)
5–10 years ago	20.0 % (9)
>10 years ago	13.3 % (6)

**Cancer stage at diagnosis**	

Stage 0	2.2 % (1)
Stage 1	15.6 % (7)
Stage 2	44.4 % (20)
Stage 3	33.3 % (15)
Unknown	4.4 % (2)

**Treatment end**	

6–12 months ago	15.6 % (7)
1–2 years ago	28.9 % (13)
2–5 years ago	26.7 % (12)
5–10 years ago	20.0 % (9)
>10 years ago	8.9 % (4)

**Treatment**	

Chemotherapy	100.0 % (45)
Hormonal therapy	60.0 % (27)
Radiation therapy	80.0 % (36)
Surgery	100.0 % (45)
Immunotherapy	4.4 % (2)
Targeted therapy	17.8 % (8)

**Current hormone therapy**	

Yes	53.3 % (24)
No	6.7 % (3)
Not applicable	40.0 % (18)

The timeframes were determined based on the day of study participation.

### Procedure

**Figure 1:**
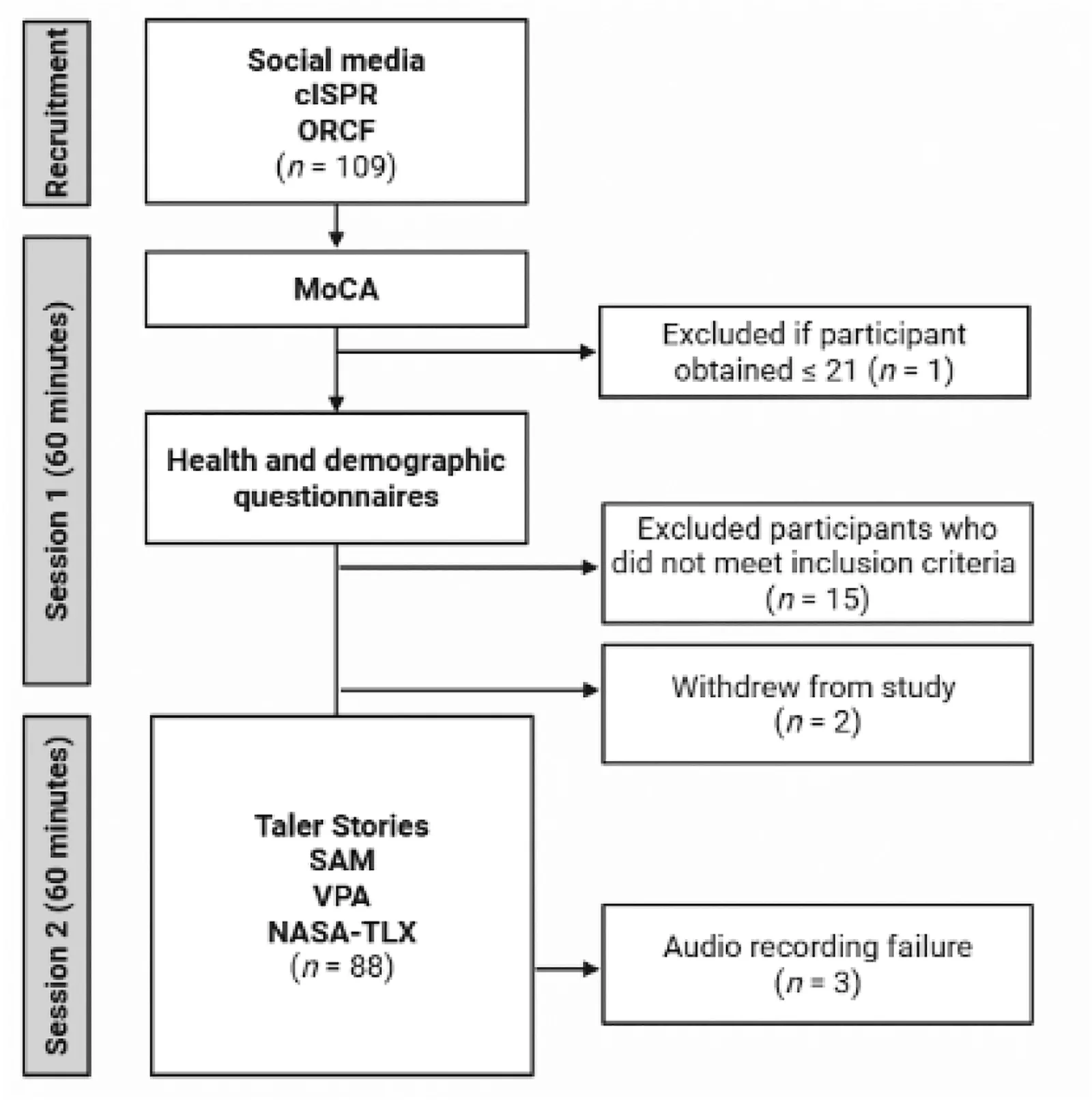
Schematic of the study design. cISPR, Community Integrated System of Participation in Research at the University of Ottawa; DR, Delayed Recall; MoCA, Montreal Cognitive Assessment; IR, immediate recall; n, sample size; NASA-TLX, National Aeronautics and Space Administration Task Load Index; ORCF, Ottawa Regional Cancer Foundation; SAM, Survey of Autobiographical Memory; VPA, Verbal Paired Associates.

This study involved two online sessions, each lasting up to 60 min (Figure 1). In session one, participants completed the videoconference version of the MoCA. Eligible participants then filled out a sociodemographic and health questionnaire via Qualtrics (RRID: SCR_016728), an online questionnaire tool. In session two, participants began with the immediate recall of two Taler stories [37], followed by 20 min of various cognitive measures (omitted from this paper), including the Survey of Autobiographical Memory (SAM; [47]), and then performed the delayed recall of the Taler Stories and the VPA [48] on Gorilla (RRID: SCR_020991), an online research platform. The National Aeronautics and Space Administration Task Load Index (NASA-TLX) was completed after each task, except the SAM [49]. All tasks were adapted for online administration, with auditory stimuli pre-recorded and participants’ spoken responses recorded via Gorilla. Throughout, participants remained connected on Zoom, with a research team member available for technical support.

### Measures

#### Montreal cognitive assessment (MoCA)

The MoCA is a 13-item cognitive screening tool designed to detect mild cognitive impairment by evaluating memory, attention, language, executive functioning, and visuospatial abilities [46]. Scores from each item are summed to yield a total score on 30, with lower scores indicating greater cognitive impairment. A cut-off score of ≤21 was applied for this study, as the standard cut-off of 26 may lead to over-pathologizing cognitive difficulties in this clinical population [50].

#### Taler stories

The Taler Stories task is designed to assess episodic verbal memory [37] and was developed as an alternative to WMS – LM [51] to refine scoring criteria, enhance interpretation of group differences, and improve inter-rater reliability [37]. For this study, one pair from the 12 Taler Stories pairs was randomly selected. Participants listened to two stories, each comprised of four sentences totaling 65 to 69 words, then recalled them immediately and again after a 20-min delay. Responses were scored as verbatim (exact word-for-word recall), gist (accurate, rephrased content), or distortion (inaccurate details). For example, “*One sunny Saturday, Fred and Grace went to visit their two grandchildren in New York*”, would be scored as: “went to visit” is verbatim, “went to see” is gist, and “went to pick up” is a distortion. Each story contains 50 informational units, which are scored separately to generate verbatim, gist, and distortion scores – each with a maximum of 50 points. A high verbatim score reflects greater recall of episodically rich and specific details, a high gist score indicates accurate recall of the story’s general content and themes, whereas a high distortion score reflects inaccurate recall of story content. A retrieval success score was calculated by summing verbatim and gist scores and subtracting distortions to capture total accurate recall while accounting for errors.

#### Verbal paired associates (VPA)

The VPA task is a widely used measure of verbal episodic memory [48]. Participants listened to a list of eight unrelated word pairs (e.g., banana-shoe). Afterward, they heard one word from each pair (e.g., banana) and were asked to recall the corresponding target word (e.g., shoe). The procedure was repeated across four trials, with the stimuli presented in a fixed, trial-specific sequence. The total score out of 32 was derived by summing the correct responses for each word pair across all four trials, with lower scores indicating worse episodic recall.

#### Survey of autobiographical memory (SAM)

The SAM is a 26-item self-report questionnaire designed to assess perceived abilities related to episodic, semantic, future, and spatial memory [47]. Participants rated each item using a 4-point Likert scale (strongly disagree to strongly agree). Each item response was converted to a numerical score based on an established scoring procedure [47]. Domain-specific scores were calculated by summing relevant items, with higher scores indicating better perceived memory functioning. For this study, only episodic and semantic subscales were analyzed.

#### National aeronautics and space administration task load (NASA-TLX)

The NASA-TLX is a self-report measure designed to assess cognitive workload during tasks [49]. The six-item questionnaire evaluates various domains of workload: mental demand, physical demand (omitted from this study), temporal demand, performance, effort, and frustration. Participants rated each item on a 20-point scale, which was summed to yield a maximum score of 100, with higher scores indicating higher perceived cognitive workload.

### Statistical analysis

Responses were manually transcribed, scored, and double-checked by study researchers blind to conditions. Inter-rater reliability for the Taler Stories scoring exceeded 90 %, with scoring discrepancies resolved through discussion. Statistical analyses were conducted using SPSS 28 (RRID:SCR_002865). Four 2 × 2 repeated measures ANOVAs (RM ANOVA) assessed four memory recall types on the Taler Stories (verbatim, gist, distortions, retrieval success) across time (immediate vs. delayed) for both groups (BCS vs. NC). Bonferroni correction for multiple comparisons was applied when necessary to reduce the risk of type I errors. Independent samples t-tests compared group performance on the VPA, SAM, and NASA-TLX. Pearson correlations (r) assessed the relationship between: NASA-TLX and Taler Stories – Immediate Recall, NASA-TLX and Taler Stories – Delayed Recall, and NASA-TLX and VPA. Statistical significance was set at α=0.05, and partial eta squared (ηp^2^) was calculated to estimate effect sizes. Figures were generated using GraphPad Prism (RRID:SCR_002798). Final sample sizes varied by task due to missing data: Taler Stories (n=83; BCS=42, NC=41), SAM (n=87; BCS=45, NC=42), and VPA (n=88; BCS=45, NC=43).

## Results

### Taler stories

**Figure 2:**
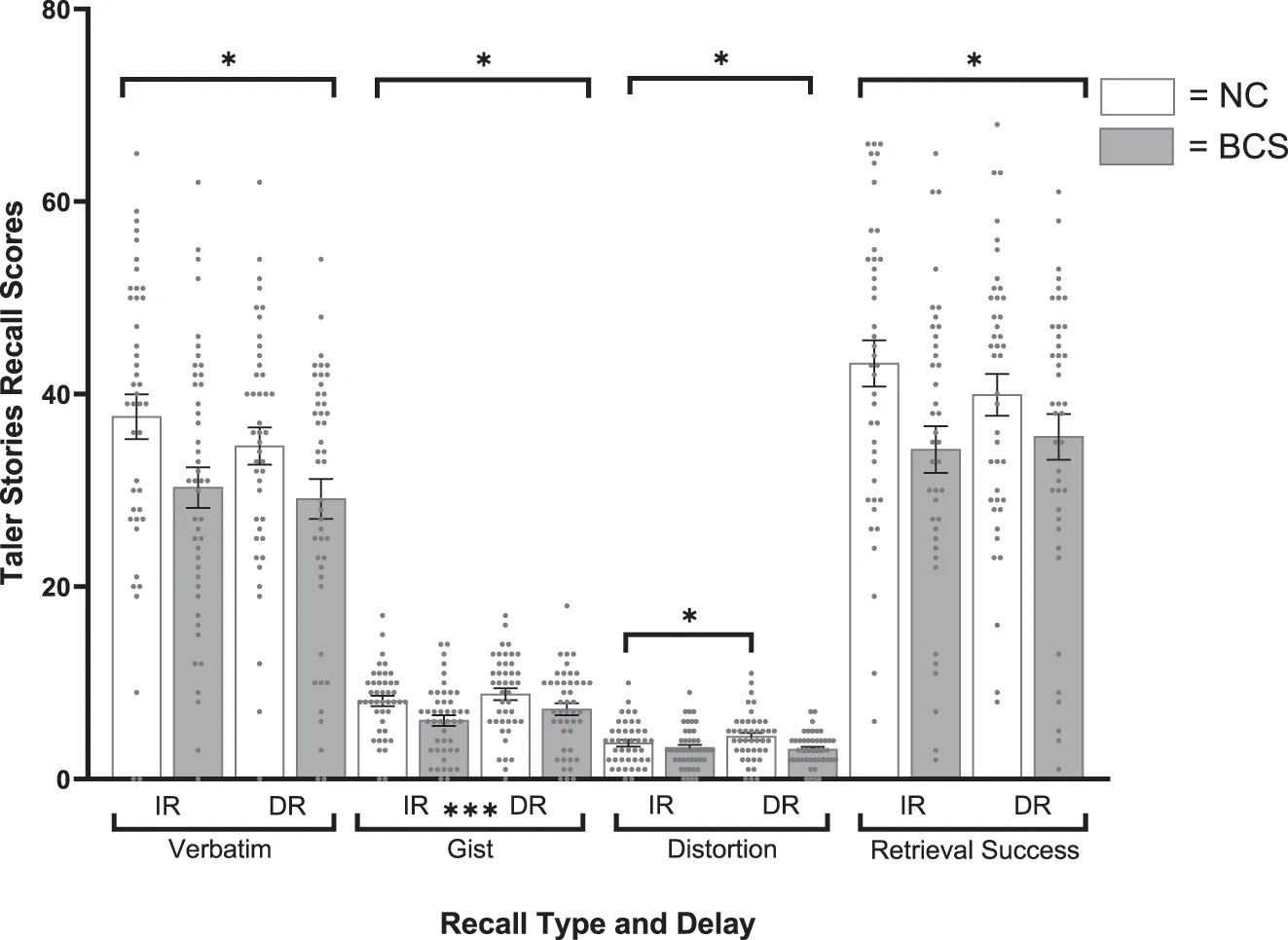
Breast cancer survivors and non-cancer controls performance on the Taler Stories. Grey bars represent breast cancer survivors, white bars represent non-cancer controls, error bars indicate the standard error of the mean (SEM), and individual data points are overlaid in light grey. Significant stars at the top represent main effects of group, in the middle represents a significant interaction, and between IR and DR represents a main effect of time. *denotes p<0.05 level and ***denotes p<0.001. Abbreviations: BCS, breast cancer survivor; NC, non-cancer controls; IR, immediate recall for Taler Stories; DR, delayed recall for Taler Stories.

Verbatim: RM ANOVA revealed a main effect of group (*F*_1,81_=5.88, p=0.02, ηp^2^=0.06), with BCS recalling fewer verbatim details than NC. No main effect of time (*F*_1,81_=3.48, p=0.06, ηp^2^=0.04) or time X group interaction (*F*_1,81_=1.85, p=0.17, ηp^2^=0.02) were observed (Figure 2).

Gist: A main effect of group (*F*_1,81_=5.82, p=0.02, ηp^2^=0.06) and time (*F*_1,81_=12.31, p<0.001, ηp^2^=0.13) emerged, with BCS reporting fewer gist details relative to NC, and both groups reporting more gist details during delayed recall compared to immediate recall. No time X group interaction was observed (*F*_1,81_=0.49, p=0.48, ηp^2^=0.006; Figure 2).

Distortion: A main effect of group (*F*_1,81_=4.08, p=0.04, ηp^2^=0.04), with BCS reporting fewer distortions relative to NC, and a group X time interaction (*F*_1,81_=4.24, p=0.04, ηp^2^=0.05) was observed. Post-hoc analyses identified more distortions committed by NC relative to BCS during delayed recall (p=0.008), but similar performance during immediate recall (p=0.34). Additionally, NC committed significantly more distortions over time (p=0.02), whereas BCS showed no time-related difference (p=0.61). No main effect of time (*F*_1,81_=1.75, p=0.18, ηp^2^=0.02) was observed (Figure 2).

Retrieval success: A main effect of group (*F*_1,81_=5.58, p=0.02, ηp^2^=0.06) revealed that BCS reported fewer total correct story details compared to NC. No main effect of time (*F*_1,81_=0.48, p=0.49, ηp^2^=0.006) or time X group interaction (*F*_1,81_=–3,65, p=0.06, ηp^2^=0.04) were observed (Figure 2).

### Verbal paired associates

**Figure 3:**
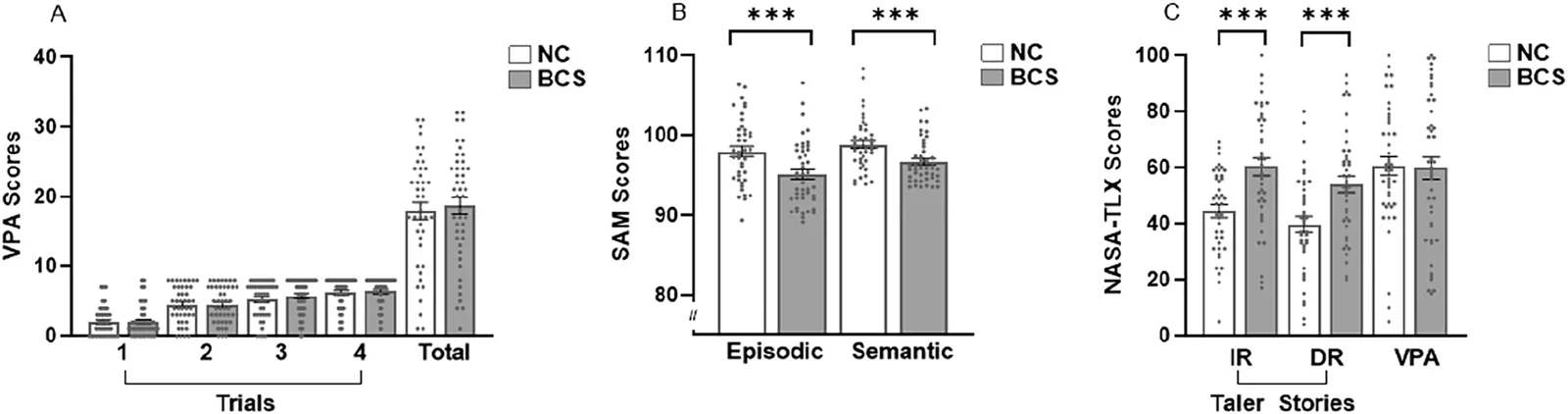
Breast cancer survivors and non-cancer controls performance on the cognitive measures. (A) Verbal paired associates (VPA), (B) survey of autobiographical memory (SAM) and (C) national aeronautics and space administration task load index (NASA-TLX). Grey bars represent breast cancer survivors, white bars represent non-cancer controls, error bars indicate the standard error of the mean (SEM), and individual data points are overlaid in light grey. ***denotes p<0.001. Abbreviations: BCS, breast cancer survivor; NC, non-cancer controls; IR, immediate recall for Taler Stories; DR, delayed recall for Taler Stories; NASA-TLX, National Aeronautics and Space Administration Task Load Index; SAM, Survey of Autobiographical Memory; VPA, Verbal Paired Associates.

An independent sample t-test identified comparable VPA memory between groups (*t*_86_=0.41, p=0.34). No group differences were observed within each of the four individual VPA trials (all p’s>0.05; Figure 3A).

### Survey of autobiographical memory

BCS rated higher subjective difficulties with their episodic (*t*_85_=−3.25, p<0.001) and semantic (*t*_85_=−3.38, p<0.001) memory abilities compared to NC (Figure 3B).

### Cognitive workload

**Figure 4:**
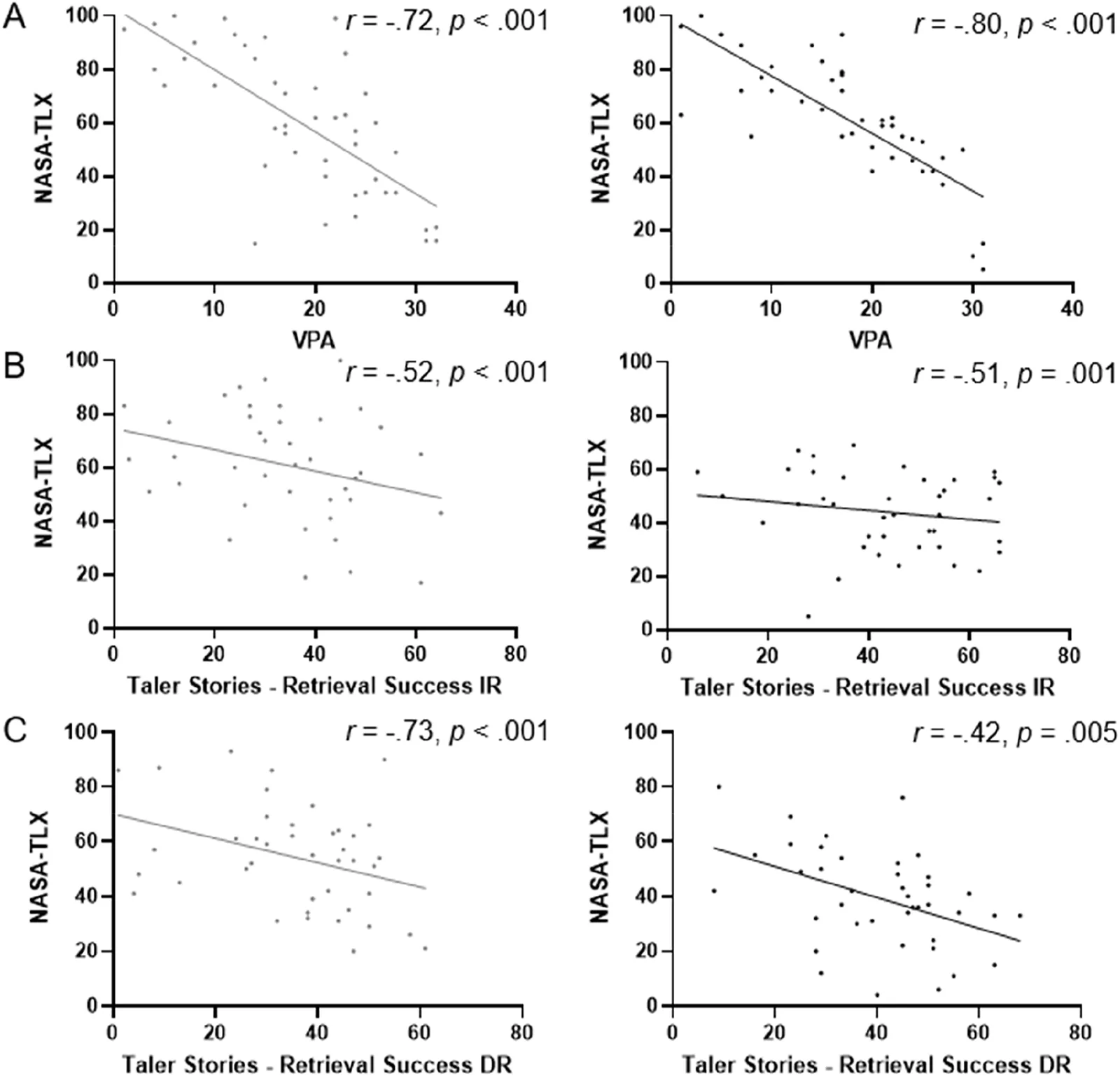
Correlations. (A) NASA-TLX and VPA, (B) NASA-TLX and Taler Stories Successful Retrieval for Immediate Recall, (C) NASA-TLX and Taler Stories Successful Retrieval for Delayed Recall.

BCS reported more cognitive effort than NC during both the immediate (*t*_81_=4.02, p<0.001) and delayed (*t*_81_=3.52, p<0.001) recall phases of the Taler Stories task (Figure 3C). However, comparable ratings of cognitive effort were reported by both groups for the VPA task (*t*_86_=−0.14, p=0.44; Figure 3C). Correlational analyses revealed higher cognitive workload was associated with poorer task performance on the following: VPA (BCS, r_43_=−0.72, p=0.005; NC, r_41_=−0.80, p<0.001; Figure 4A); successful retrieval during Taler Stories immediate recall (BCS, r_40_=−0.52, p<0.001; NC, r_39_=−0.51, p=0.001; Figure 4B); successful retrieval during Taler Stories delayed recall (BCS, r_40_=−0.73, p<0.001; NC, r_39_=−0.42, p=0.005; Figure 4C).

## Discussion

This study examined the long-term effects of cancer treatment on episodic memory in BCS, using both narrative and standardized VPA tasks. Uniquely, it is the first to assess recall of verbatim, gist, and distortion details of narrative event memories in this population. As hypothesized, BCS showed impaired performance on the narrative memory task and reported greater cognitive workload compared to NC. In contrast, their performance on the VPA task was comparable to that of NC, with no reported increase in cognitive workload to complete this task. These findings were contrary to our expectations, and highlight an important limitation to standardized memory tasks that may contribute to an under-reporting of cognitive impairment following cancer treatment.

BCS recalled fewer verbatim and gist-based details on a naturalistic memory task compared to NC. Despite these differences, both groups performed similarly across immediate and delayed recall, suggesting similar patterns of forgetting even over a brief period of 20 min. These findings are noteworthy given that verbatim memory typically declines over time, even in healthy individuals [52]. Both groups recalled more gist details after a delay, consistent with memory becoming more gist-like and schematic over time [12], a finding that is also observed over longer periods of weeks and months. An unexpected finding was survivors reporting fewer gist details than NC, potentially indicating generally less accessible memory content [53]. Interestingly, only NC had a significant increase in memory distortions over time. These results support prior findings of cancer survivors recalling fewer narrative details [29], 54], 55], but contrast with studies showing no effects of chemotherapy [31], 56]. Accounting for memory distortions, BCS recalled fewer accurate gist and verbatim details than NC – a difference that remained consistent over time.

The present findings align with those of Taler et al. [37] who developed this narrative task to assess episodic memory quality in both healthy aging and in cases of mild cognitive impairment (MCI). Our results indicate that both BCS and NC groups reported fewer verbatim details following the delay, mirroring the pattern observed by Taler et al. when comparing healthy younger and older adults. Overall, younger adults produced more verbatim details than older adults, who in turn outperformed individuals with MCI. This pattern parallels our observation that NC participants reported more verbatim details than BCS participants, which potentially suggests that BCS may follow an accelerated trajectory of age-related memory changes, mirroring patterns seen in typical aging [57].

The finding that NC reported more memory distortions relative to BCS is counterintuitive but identifies an interesting distinction. Rather than reflecting superior memory accuracy, the lower distortion and gist scores among BCS likely indicate impairments in memory encoding [29], 31]. While memory naturally transforms over time, becoming less specific and more prone to distortion particularly for schema-consistent events, BCS may encode fewer details from the outset, leaving less information available for later recall or distortion [11], 12], 18]. This interpretation is reinforced by lower overall recall and supported by retrieval success scores, which account for both accuracy and completeness of memory.

In contrast to the narrative memory task, BCS and NC performed similarly on the VPA. This suggests that certain aspects of episodic memory, particularly simple associative learning, may remain relatively preserved following treatment. These findings align with prior findings that VPA performance lacks the sensitivity to detect subtle cognitive deficits, even in individuals with MCI [58].

BCS reported greater cognitive effort than NC during narrative recall, but not during the VPA task, suggesting that more complex, real-world memory tasks place higher cognitive demands on BCS. This pattern implies that BCS may recruit additional cognitive resources through compensatory mechanisms to perform narrative recall tasks [59], 60]. However, across both groups, higher subjective cognitive effort was associated with worse memory performance, indicating that these impressions likely reflect awareness of cognitive challenges [32]. BCS also reported greater subjective difficulties with both episodic and semantic memory, consistent with previous research identifying persistent perceived memory problems following treatment [61], 62].

Methodological variability likely contributes to inconsistencies in the literature on chemotherapy-related cognitive impairment, including differences in participants, treatment type and dosage, time since treatment, and measures. While many studies find that chemotherapy-treated cancer survivors perform worse on neuropsychological tests and report more memory and concentration difficulties, these subjective complaints often correlate more strongly with psychological distress than with objective impairment [38], 63]. Additionally, survivors who had above-average cognitive abilities before treatment may still score within normal ranges post-treatment but feel they have declined compared to their personal baseline. Thus, their concerns might reflect real changes that are not captured by standard population-based comparisons [38].

Both perceived and objective cognitive difficulties should be routinely addressed throughout cancer survivorship, with regular screening across different stages of recovery [64], 65]. Although clinical resources may be limited, comprehensive pre- and post-treatment neuropsychological assessments that incorporate standardized and ecologically valid measures, and subjective measures of cognitive functioning are recommended to define cognitive profiles and guide interventions [62], [66], [67], [68]. This ongoing approach can help validate patient experiences and support appropriate management of cognitive challenges over time [64].

## Limitations

Our online cross-sectional design enabled recruitment of a geographically diverse Canadian sample, including immunocompromised participants. However, reliance on social media recruitment may have biased the sample toward younger, Caucasian groups [69], 70], resulting in limited diversity and reduced generalizability. Computerized cognitive assessments allow for standardized administration, enhanced accessibility, feasibility, and participant comfort in a familiar environment [71], 72]. Although this can introduce variability related to environment, fatigue, distractions, and technology [71], 72], this was mitigated by conducting sessions via Zoom with a trained researcher present throughout the study. Additionally, although treatment-related factors are known to influence cognition [3], unavailable or incomplete patient-reported treatment details prevented covariate analyses, limiting considerations of their influence on cognitive outcomes. Future research should collect detailed medical data to clarify mechanisms underlying cancer-related cognitive dysfunction. Additionally, longitudinal design that include pre-treatment cognitive and neuroimaging assessments would enable stronger causal inferences about the emergence and development of treatment-related cognitive and neural changes.

## Conclusions

This study adds to evidence of persistent cognitive challenges among BCS, specifically in episodic memory. By contrasting a naturalistic narrative task with a standardized memory task, we show that traditional assessments may overlook real-world memory difficulties. Ecologically valid measures that better reflect survivors’ lived experiences paired with a cognitive workload measure provide a more nuanced view of both memory function and the subjective effort required to complete everyday tasks. These results underscore the need for multidimensional cognitive assessments in survivorship care and support the development of tailored interventions that validate survivors’ experiences and enhance quality of life.
